# From pathophysiology to practice: addressing oxidative stress and sperm DNA fragmentation in Varicocele-affected subfertile men

**DOI:** 10.1590/S1677-5538.IBJU.2024.9917

**Published:** 2024-07-25

**Authors:** Filipe Tenório Lira, Lucas Ribeiro Campos, Matheus Roque, Sandro C. Esteves

**Affiliations:** 1 Andros Recife Recife PE Brasil Andros Recife, Recife, PE, Brasil; 2 Instituto de Medicina Integral Prof. Fernando Figueira Departamento de Urologia Recife PE Brasil Departamento de Urologia, Instituto de Medicina Integral Prof. Fernando Figueira, Recife, PE, Brasil; 3 Hospital Santa Joana Recife Recife Brasil Hospital Santa Joana Recife, Recife, PE, Brasil; 4 Universidade Federal de Minas Gerais Departamento de Urologia Belo Horizonte MG Brasil Departamento de Urologia, Universidade Federal de Minas Gerais (UFMG), Belo Horizonte, MG, Brasil; 5 Mater Prime Departamento de Medicina Reprodutiva São Paulo SP Brasil Departamento de Medicina Reprodutiva, Mater Prime, São Paulo, SP, Brasil; 6 ANDROFERT - Clínica de Andrologia e Reprodução Humana Centro de Referência. em Reprodução Masculina Campinas SP Brasil ANDROFERT - Clínica de Andrologia e Reprodução Humana, Centro de Referência. em Reprodução Masculina, Campinas, SP, Brasil; 7 Disciplina de Urologia da Universidade Estadual de Campinas Departamento de Cirurgia Campinas SP Brasil Departamento de Cirurgia, Disciplina de Urologia da Universidade Estadual de Campinas - UNICAMP, Campinas, SP, Brasil; 8 Aarhus University Faculty of Health Department of Clinical Medicine Aarhus Denmark Department of Clinical Medicine, Faculty of Health, Aarhus University, Aarhus, Denmark

**Keywords:** Infertility, Male, Oxidative Stress, Varicocele, Reproductive Techniques, Assisted

## Abstract

Varicocele can reduce male fertility potential through various oxidative stress mechanisms. Excessive production of reactive oxygen species may overwhelm the sperm's defenses against oxidative stress, damaging the sperm chromatin. Sperm DNA fragmentation, in the form of DNA strand breaks, is recognized as a consequence of the oxidative stress cascade and is commonly found in the ejaculates of men with varicocele and fertility issues. This paper reviews the current knowledge regarding the association between varicocele, oxidative stress, sperm DNA fragmentation, and male infertility, and examines the role of varicocele repair in alleviating oxidative-sperm DNA fragmentation in these patients. Additionally, we highlight areas for further research to address knowledge gaps relevant to clinical practice.

## INTRODUCTION

Varicocele is the abnormal enlargement of the veins within the pampiniform plexus due to venous blood reflux caused by incompetent venous valves (1, 2). The condition affects around 15% of the adult male population and 35% of men with primary infertility (3-5). Its incidence rises to 80% among men with secondary infertility, suggesting progressive damage of spermatogenesis (3). This hypothesis is further supported by the progressive impairment of semen analysis (SA) parameters in men with untreated varicocele (6). Most studies involving infertile men with varicocele have shown that the condition impairs SA parameters, such as sperm concentration, sperm motility, and sperm morphology (7-9). Conversely, it is estimated that 80% of the men with varicocele are fertile, making the association between varicocele and decreased semen quality controversial in fertile males (7-10).

Traditionally, the damage to reproductive function caused by varicocele is attributed to testicular hyperthermia due to the loss of the countercurrent mechanism that keeps the testicular temperature 2oC below the core temperature (11). However, recent studies have demonstrated that several non-mutually exclusive factors, including excessive oxidative stress (OS), are implicated in the pathophysiology of varicocele (1).

Currently, varicocele repair is recommended for infertile men with clinical varicocele and abnormal basic SA parameters (12). An abnormal basic semen analysis is defined by alterations in classic parameters like sperm concentration, total sperm count, total motility, progressive motility, normal forms, and vitality (13). Clinical varicocele is defined as a dilation of the pampiniform plexus, either palpable or visible during the physical examination with the patient standing (14). Varicoceles are graded using the criteria of Dubin and Amelar as absent - no palpable varicocele, grade 1 – palpable only with Valsalva maneuver, grade 2 - palpable without Valsalva, and grade 3 - visible (15). The term "subclinical varicocele" is used when the varicocele is not palpable, even with the Valsalva maneuver, but detected by imaging studies, such as the color Doppler scrotal ultrasound (16). Based on these definitions, the treatment of clinical varicoceles in infertile men has been consistently associated with SA parameters, reduced oxidative stress, higher pregnancy rates, and better outcomes in assisted reproductive technology (ART) (4, 17-20). Additionally, improved reproductive outcomes after varicocelectomy have been linked to reduced OS and sperm DNA fragmentation (21, 22, 23), suggesting that elevated sperm DNA fragmentation levels should be an indication for varicocele repair (24).

In this paper, we review the current knowledge regarding the association between varicocele, oxidative stress, sperm DNA fragmentation, and male infertility, and examine the role of varicocele repair in alleviating oxidative-sperm DNA fragmentation in these patients. We also highlight areas for further research to address knowledge gaps relevant to clinical practice.

### Mechanisms of Varicocele-Induced Oxidative Stress

Reactive oxygen species (ROS) are reactive chemical intermediates with one or more unpaired electrons that quickly react with organic compounds to stabilize their electronic structures (25). While primarily viewed as toxic agents, ROS are necessary for normal sperm function. A small degree of lipid peroxidization of the sperm membrane enhances the ability of sperm to bind to the zona pellucida (26). Additionally, small amounts of superoxide, the primary free radical, have been shown to induce hyperactivation and capacitation of human sperm (27). However, OS occurs when excessive ROS are produced, surpassing the antioxidant mechanisms. Unchecked lipid peroxidation and other reduction reactions cause alterations in nuclear and mitochondrial sperm DNA, such as base modification, strand breaks, and chromatin cross-links (28, 29). Due to the limited capacity of spermatozoa to repair its membrane and genetic material, these damages ultimately lead to apoptosis and defective sperm function (30-34).

ROS can be measured using direct or indirect methods (35). Indirect techniques assess by-products of oxidation, such as lipid peroxidation (MDA), protein oxidation (like protein carbonyl), and oxidized DNA (8-hydroxy-2′-deoxyguanosine[8-OHdG]). Direct oxidative stress measurements include total or specific ROS levels in semen and total antioxidant capacity (TAC) (36).

Varicocele has been consistently associated with OS and decreased seminal antioxidant capacity (Table-1). This association is more substantial when comparing infertile men with varicocele to fertile men without varicocele (37-46), but even fertile men with varicocele have increased levels of ROS (47-50). Seminal OS biomarkers are found in higher concentrations in infertile men with varicocele, regardless of alterations in basic SA parameters (38, 51). A study by Gill and colleagues involving infertile men with clinical varicocele reported that 83% of these men have elevated OS, measured by oxidation-reduction potential (ORP> 1.37 mV/106 sperm/mL), significantly higher compared to 19% of the men with proven fertility (P < 0.05) (46). Moreover, varicocele increases seminal ROS levels as early as adolescence (52).

**Table 1:** Characteristics of the studies assessing the effect of varicocele on seminal oxidative stress.

Author, year, (country)	Assays	Study groups	Main results
Hendin et al., 1999 (USA) (49)	Seminal ROS by chemiluminescence with luminol; seminal TAC by enhanced chemiluminescence	17 normozoospermic men without varicocele; 15 men with incidental varicocele; 21 infertile men with palpable varicocele	**ROS levels:** Controls: 1.3 ± 0.33 log[ROS+1] Men with incidental varicocele: 1.99 ± 0.26 log[ROS+1] (P<0.05 *versus* controls); Infertile men with varicocele:2.18 ± 0.25 log[ROS+1] (P<0.05 *versus* controls); **TAC:** Controls: 1443.0 ± 105.0 molar Trolox; Men with incidental varicocele: 939.0 ± 107.0 molar Trolox (P<0.05 *versus* controls); Infertile men with varicocele: 1186.0 ± 96.9 molar Trolox (P<0.05 *versus* controls)
Sharma et al., 1999 (USA) (43)	Seminal ROS by chemiluminescence with luminol; seminal TAC by enhanced chemiluminescence	25 normozoospermic fertile, healthy men; 55 infertile men with palpable varicocele	**ROS levels:** Controls: 1.39 ± 0.73 log[ROS+1]; Infertile men with varicocele: 2.10 ± 1.21 log[ROS+1] (P<0.05 *versus* controls) **TAC:** Controls: 1650.93 ± 532.22 molar Trolox; Infertile men with varicocele: 1100.11 ± 410.13 molar Trolox (P<0.05 *versus* controls)
Köksal et al., 2000 (Turkey) (56)	Intratesticular MDA by thiobarbituric acid reaction	10 infertile men without varicocele; 15 infertile men with palpable varicocele	**MDA levels:** Infertile men without varicocele: 33.5 ± 18.93 pmol/mg; Infertile men with varicocele: 38.3 ± 22.92 pmol/mg (P NS *versus* controls); MDA levels in men with grade III varicocele were higher than in men with lower grade varicocele (P<0.05)
Pasqualotto et al., 2000 (USA) (40)	Seminal ROS by chemiluminescence with luminol; Seminal TAC by enhanced chemiluminescence	19 normozoospermic men without varicocele; 77 infertile men with palpable varicocele	**ROS levels:** Controls: 1.3 ± 0.3 log[ROS+1]; Infertile men with varicocele: 2.2 ± 0.13 log[ROS+1] (P<0.05 *versus* controls) **TAC:** Controls: 1653.98 ± 115.29 molar Trolox; Infertile men with varicocele: 1173.05 ± 58.07 molar Trolox (P<0.05 *versus* controls)
Saleh et al., 2003 (USA) (44)	Seminal ROS by chemiluminescence with luminol; Seminal TAC by enhanced chemiluminescence	16 fertile men without varicocele; 15 infertile men without varicocele; 16 infertile men with palpable varicocele	**ROS levels:** Controls: 0.36 (IQR: 0.1, 2) (cpm)/20×106 sperm/mL; Infertile men without varicocele: 1.7 (IQR: 0.1, 5.4) (cpm)/20×106 sperm/mL (P NS *versus* controls); Infertile men with varicocele: 12 (IQR: 1.3, 53.4) (cpm)/20×106 sperm/mL (P<0.05 versus controls) **TAC:** Controls: 871 (IQR: 699, 1288) molar Trolox; Infertile men without varicocele: 904 (IQR: 693, 978) molar Trolox (P NS *versus* controls); Infertile men with varicocele: 693 (IQR: 499, 822) molar Trolox (P<0.05 *versus* controls)
Allamaneni et al., 2004 (USA) (57)	Seminal ROS by chemiluminescence with luminol; Seminal TAC by enhanced chemiluminescence	46 infertile men with palpable left varicocele	Median ROS level 119 (13, 2475) x10^4^cpm ROS levels positively correlated with varicocele grade
Mehraban et al., 2005 (Iran) (37)	Seminal total nitrite and nitrate levels	40 fertile men without varicocele; 40 infertile men without varicocele; 40 infertile men with palpable varicocele	Seminal total nitrite and nitrate levels: Controls: 37.06 ± 20.39 μmol/L; Infertile men without varicocele: 33.7 ± 18.99 μmol/L (P NS *versus* controls); Infertile men with varicocele: 52.34 ± 26.62 μmol/L (P<0.05 versus controls and infertile men without varicocele)
Smith et al., 2006 (Chile) (98)	Seminal ROS by chemiluminescence with luminol; Seminal TAC by enhanced chemiluminescence	25 normozoospermic healthy donors 37 men with varicocele and normal SA; 18 men with varicocele and abnormal SA	**ROS levels:** Controls: 2.8 ± 0.9 log[ROS+1]; Men with varicocele and normal SA: 3.3 ± 1.2 log[ROS+1] (P<0.05 *versus* controls); Men with varicocele and abnormal SA: 4.3 ± 1.1 log[ROS+1] (P<0.05 *versus* controls) **TAC:** Controls: 1.2 ± 0.1 mM Trolox; Men with varicocele and normal SA: 1.1 ± 0.4 mM Trolox (P NS *versus* controls); Men with varicocele and abnormal SA: 1.1 ± 0.5 mM Trolox (P NS *versus* controls)
Ishikawa et al. 2007 (Japan) (58)	Intratesticular 8-OHdG positive cell by immunostaining	5 healthy fertile men; 36 infertile men with palpable varicocele and abnormal SA	Incidence of 8-OHdG immunostained germ cells: Controls: 29 ± 5.4%; Varicocele grade I:38 ±10%, (P<0.05 *versus* controls); Varicocele grade II: 41 ± 9.1% (P<0.05 *versus* controls); Varicocele grade III: 57 ± 9.3% (P<0.05 versus controls and grade I+II)
Sakamoto et al., 2008 (Japan) (38)	Seminal NO levels; Seminal 8-OHdG levels; Seminal SOD activity	15 normozoospermic men without varicocele; 15 infertile men with varicocele and normal SA; 15 infertile men with palpable varicocele and oligozoospermia	**NO levels:** Controls: 8.2 ± 4.3μmol/L; Men with varicocele and normal SA:15.4 ± 0.3μmol/L (P<0.05 *versus* controls); Men with varicocele and oligozoospermia: 7.8 ± 4.0 μmol/L (P NS versus controls); **8-OHdG levels:** Controls: 14.7 ± 8.3 μmol/L; Men with varicocele and normal SA:10.0 ± 5.4 μmol/L (P NS *versus* controls); Men with varicocele and oligozoospermia: 10.8 ± 7.5 μmol/L (P NS *versus* controls); **SOD activity:** Controls: 75.6 ± 13.1%; Men with varicocele and normal SA: 84 ± 6.7% (P<0.05 *versus* controls); Men with varicocele and oligozoospermia: 89.4 ± 4.4 % (P<0.05 *versus* controls)
Mostafa et al., 2009 (Egypt) (47)	Seminal MDA by thiobarbituric acid reaction; Seminal H2O2 by spectrophotometric Method; Seminal SOD; Seminal GPx; Seminal Cat	45 fertile men without varicocele; 45 fertile men with varicocele; 42 infertile men with unilateral palpable varicocele and abnormal SA; 44 infertile men with abnormal SA and without varicocele	MDA and H2O2 were significantly higher, and antioxidants were significantly lower in fertile men with varicocele, OA men with and without varicocele compared with controls; All ROS parameters were increased, and all antioxidants were decreased in infertile men with varicocele compared to infertile men without varicocele
Abd-Elmoaty et al., 2010 (Egypt) (62)	Seminal NO levels by colorimetric method; Seminal MDA by colorimetric method; Seminal SOD; Seminal GPx; Seminal Cat	18 fertile men without varicocele; 42 infertile men with varicocele	**MDA levels:** Controls: 8.4 ± 1.3 pmol/mL; Infertile men with varicocele: 13.5 ± 2.8 pmol/mL (P<0.05 *versus* controls) **NO levels:** Controls: 11.3 ± 1.0 nmol/L; Infertile men with varicocele: 17.9 ± 4.1 nmol/L (P<0.05 *versus* controls); CAT, SOD, GPX, and ascorbic acid were significantly lower in infertile men with varicocele compared with fertile men (P values <.05, .01, .01, and .05, respectively)
Blumer et al., 2011 (Brazil) (165)	Seminal MDA by thiobarbituric acid reaction	19 men without varicocele; 12 men with varicocele (fertility status not informed);	**MDA levels:** Controls: 301.4 ± 95.9 ng/mL Men with varicoceles: 287.1 ± 127.7 ng/mL (P NS *versus* controls)
Mostafa et al., 2012 (Egypt) (59)	Seminal MDA by thiobarbituric acid reaction; Seminal H2O2 by spectrophotometric Method; Seminal SOD; Seminal GPx; Seminal Cat	20 fertile men without varicocele; 22 infertile men with grade 1varicocele; 43 infertile men with grade II varicocele; 23 infertile men with grade III varicocele	Levels of MDA and H2O2 were increased and antioxidants; SOD, Cat, GPx, vit. C levels were decreased in men with varicocele of all grades (I, II, III) compared with the controls; Men with grade II and III varicocele demonstrated higher MDA and H2O2 levels as well as decreased activities of SOD, Cat, GPx, and levels of vit. C compared with men with grade I varicocele.
Mostafa et al., 2016 (Egypt) (50)	Seminal MDA by thiobarbituric acid reaction; Seminal GPx	24 fertile men without varicocele; 22 fertile men with varicocele; 34 infertile men with palpable varicocele and abnormal SA; 24 infertile men with abnormal SA and without varicocele	**MDA levels:** Controls: 1.2 ± 0.17 nmol/mL; Fertile men with varicocele: 1.9 ± 0.69 nmol/mL (P<0.05 *versus* controls); Infertile men without varicocele and abnormal SA: 2.4 ± 0.47 nmol/mL (P<0.05 *versus* controls and other groups); Infertile men with varicocele and abnormal SA: 3.02 ± 0.47 nmol/mL (P<0.05 *versus* controls and other groups) **GPx activity:** Controls: 0.47 ± 0.6 U/mL; Fertile men with varicocele: 0.36 ± 0.8 U/mL (P<0.05 *versus* controls); Infertile men without varicocele and abnormal SA: 0.3 ± 0.03 U/mL (P<0.05 *versus* controls and other groups); Infertile men with varicocele and abnormal SA: 0.21 ± 0.04 U/mL (P<0.05 *versus* controls and other groups)
Ni et al., 2016 (China) (61)	Seminal MDA by thiobarbituric acid reaction	25 healthy normozoospermic men without varicocele; 15 infertile men with subclinical varicocele; 19 infertile men with grade I varicocele; 18 infertile men with grade II varicocele; 14 infertile men with grade III varicocele	**MDA levels:** Control group: 7.45 ± 3.58 nmol/mL; Varicocele subclinical group: 7.22 ± 3.33 nmol/mL; Varicocele grade I group: 12.18 ± 4.86 nmol/mL (P<0.05 *versus* controls); Varicocele grade II group: 14.12 ± 5.42 nmol/mL (P<0.05 *versus* controls); Varicocele grade III group: 15.86 ± 6.78 nmol/mL (P<0.05 *versus* controls)
Abdelbaki et al., 2017 (Egypt) (42)	Seminal ROS by chemiluminescence with luminol; Seminal TAC by Colorimetric assay kit	20 normozoospermic fertile men without varicocele; 60 infertile men with palpable varicocele	**ROS levels:** Controls: 2.62 ± 0.8 log[ROS+1]; Infertile men with varicocele: 4.49 ± 0.9 log[ROS+1] (P<0.05 *versus* controls); **TAC:** Controls: 1.5 ± 0.5 mM Trolox; Infertile men with varicocele: 0.97 ± 0.4 mM Trolox (P<0.05 *versus* controls)
Alkan et al., 2018 (Turkey) (60)	Seminal ROS by chemiluminescence with luminol; Seminal superoxide anion by chemiluminescence with lucigenin	13 normozoospermic men without varicocele; 17 men with grade II varicocele; 17 men with grade III varicocele	**ROS levels:** Controls: 2.4 ± 0.1 log[ROS+1]; Men with grade II varicocele: 2.7 ± 0.4 log[ROS+1] (P<0.05 versus controls); Men with grade III varicocele: 3.2 ± 0.5 log[ROS+1] (P<0.05 *versus* controls and grade II group); **Superoxide anion levels:** Controls: 2.3 ± 0.2 log[ROS+1]; Men with grade II varicocele: 2.5 ± 0.3 log[ROS+1] (P<0.05 *versus* controls); Men with grade III varicocele: 3.0 ± 0.5 log[ROS+1] (P<0.05 versus controls and grade II group)
Tanaka et al., 2020 (Japan) (45)	Seminal ORP measured by MiOXSYS	102 normozoospermic men without varicocele; 138 infertile men with palpable varicocele	**ORP:** Men without varicocele: 9.82 ± 10.31 mV/10^6^ sperm/mL; Infertile men with varicocele: 16.73 ± 12.13 mV/10^6^ sperm/mL (P<0.05 *versus* controls)
Ammar et al., 2021 (Tunisia) (51)	Seminal MDA by thiobarbituric acid reaction; Seminal SOD; Seminal Cat; Seminal GPx	29 fertile men without varicocele; 11 infertile men with palpable varicocele and normal SA; 40 infertile men with palpable varicocele and abnormal SA	**MDA levels:** Controls: 0.56 ± 0.25 nmol/mL; Infertile men with varicocele and normal SA: 1.43 ± 1.2 nmol/mL (P<0.05 versus controls); Infertile men with varicocele and abnormal SA: 1.63 ± 1.38 nmol/mL (P<0.05 *versus* controls); GPx and CAT activities were decreased in both groups with varicocele, and SOD activity was decreased only in infertile men with varicocele and abnormal SA when compared to controls (P<0.05)
Camargo et al., 2021 (Brazil) (166)	Seminal MDA by thiobarbituric acid reaction	15 normozoospermic men without varicocele; 15 infertile men with grade 2 or 3 varicocele	**MDA levels:** Controls: 20.1 ± 4.59 nmol/mL Infertile men with varicocele: 21.6 ± 8.97 nmol/mL (P NS *versus* controls)
Gill et al., 2021 (Poland) (46)	Seminal ORP measured by MiOXSYS	105 normozoospermic men without varicocele; 64 men with proven fertility; 71 infertile men with clinical varicocele; 95 infertile men without varicocele	**ORP:** Normozoospermic group: 1.68 ± 0.91 mV/106 sperm/mL; Proven fertility group: 1.00 ± 0.8 mV/106 sperm/mL; Varicocele group: 36.10 ± 60.97 mV/106 sperm/mL (P < 0.05 versus normozoospermic and proven fertility);

8-OHdG: 8-hydroxy-2′-deoxyguanosine; Cat :catalase activity; GPx: glutathione peroxidase activity; H2O2: hydrogen peroxide; IQR: interquartile range; MDA: malondialdehyde; NO: Nitric oxide; NS: not significant; ORP: oxidation-reduction potential; ROS: Reactive oxygen species; SA: semen analysis; SOD: superoxide

Varicocele grade has been shown to influence the impairment of basic semen parameters (53, 54). For instance, a large cross-sectional study revealed that semen quality was significantly impaired in men with all varicocele grades, with the most severe impairment at higher grades (55). Higher-grade varicoceles are associated with higher levels of seminal ROS than smaller ones (42, 56-62). In contrast, one study evaluated the impact of subclinical varicocele and did not find increased OS marker levels compared to controls without varicocele (61). Moreover, the only study assessing the influence of varicocele laterality on the severity of OS demonstrated increased expression of cyclooxygenases in infertile men with bilateral varicocele compared to men with unilateral varicocele (50). Thus, it is reasonable to assume that varicocele grade influences the severity of varicocele-induced OS. However, the limited number of studies prevents a definitive conclusion regarding the impact of varicocele laterality and subclinical varicocele on ROS production.

**Figure 1:**
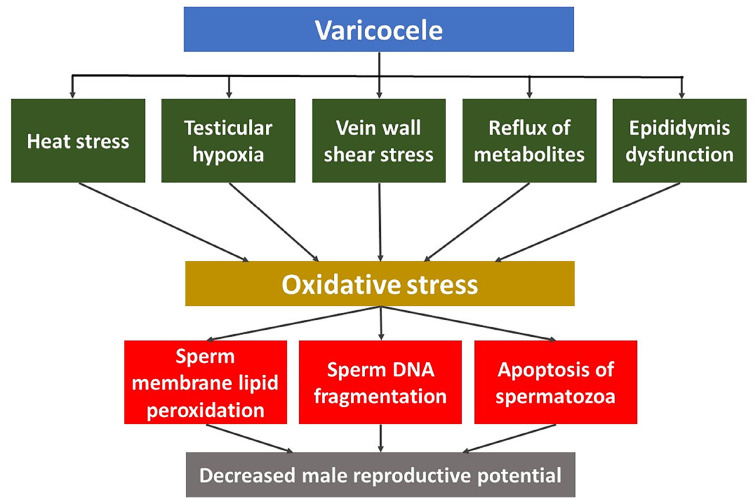
Pathophysiology of Varicocele and its Association with Sperm DNA

Despite the link between varicocele and OS, the mechanisms underpinning this association are yet to be fully clarified. The most studied effects of varicocele that could increase ROS production or decrease TAC include scrotal hyperthermia, testicular hypoxia, vein wall shear stress, adrenal/renal metabolites reflux, and epididymal response (63) (Figure-1). Additionally, most men with varicocele are fertile; however, the pathways that prevent damage to spermatogenesis in these men are unclear (10). Proposed response mechanisms include increased production of enzymatic and non-enzymatic ROS scavengers such as catalase, superoxide dismutase, vitamin C, and glutathione peroxidase (35, 48, 64).

**Fragmentation.** Lines indicate direct effects. Adapted from Cho et al. Novel insights into the pathophysiology of varicocele and its association with reactive oxygen species and sperm DNA fragmentation. *Asian J Androl*. 2016 Mar-Apr;18(2):186-93, under the Creative Commons Attribution License terms.

### Heat Stress

Scrotal hyperthermia was the first hypothesis described to explain oxidative stress in varicocele (28). The optimal temperature for spermatogenesis is 2 to 4°C lower than the body's average temperature. This difference is maintained by several mechanisms, including the contraction of the cremaster and dartos muscles and the countercurrent system in the pampiniform plexus (65). Incompetent valves of the internal spermatic and cremasteric veins allow venous blood to reflux into the pampiniform plexus, disrupting the countercurrent mechanism and raising the scrotal temperature (66, 67). Heat stress is associated with increased ROS production by several organelles, such as cell mitochondria, plasma membrane, cytoplasm, and peroxisomes (68). The severity of the damage caused by hyperthermia varies among the various cell compartments (30). In the testes, spermatogonia B and the developing spermatozoa are the most susceptible to heat stress, whereas spermatogonia A, Leydig, and Sertoli cells are relatively resistant to hyperthermia (11, 28).

### Testicular Hypoxia

Venous reflux hampers normal circulation in the testicular microvessels, leading to testicular ischemia in men with varicocele (69). Using ultra-sensitive Doppler ultrasound to measure testicular flow, Rocher and colleagues reported a decrease by 60% and 80% (P<0.05) in arterial blood flow during the Valsalva maneuver in patients with grades 2 and 3 varicoceles, respectively, suggesting that ischemia occurs when the venous hydrostatic pressure of the internal testicular vein exceeds the testicular arteriolar pressure (70). Another study demonstrated that a peak retrograde flow higher than 38 cm/s was linked to increased sperm DNA damage in men with varicocele (71). Arteriolar occlusion by microthrombi and subsequent ischemic alterations, including germ-cell degeneration, Leydig cell atrophy, and fibrotic thickening of the seminiferous tubules' basement membranes, are commonly reported in histopathological analysis of testicular biopsy specimens from infertile men with varicocele (72). This hypoxic state leads to excessive ROS generation from various molecular pathways, including activation of hypoxia-inducible factor 1 (HIF-1), mitochondrial dysfunction, xanthine dehydrogenase/oxidase, membrane-associated NADPH oxidase 5 (NOX5), and phospholipase A2 (28). Furthermore, hypoxia increases the expression of leptin and cytokines in testicular tissue, including interleukin (IL)-1 and IL-6, which also contribute to ROS production (58, 73, 74).

### Vein Wall Shear Stress

Varicose veins from patients with chronic venous insufficiency exhibit increased production of ROS and decreased antioxidant potential (75, 76). These studies suggest that the shear stress caused by local hydrostatic hypertension can activate adhesion molecules, such as selectins, integrins, intercellular adhesion molecule 1, and vascular cell adhesion protein 1, leading to increased leukocyte migration to the vein wall. Once migrated, these leukocytes become activated and produce increased amounts of ROS. The shear stress and hypoxic environment in the blood vessels can also induce excessive nitric oxide production via endothelial nitric oxide synthase, further aggravating the oxidative stress in the testicular microcirculation (77).

### Reflux of Adrenal/Renal Metabolites

Phlebographic studies have demonstrated retrograde blood flow from renal and adrenal veins to the left testicular vein in men with varicocele (78). Some authors have described the reflux of renal and adrenal metabolites, such as prostaglandins, urea, and adrenomedullin, back to the internal spermatic veins, which could induce cellular OS (79-83).

### Epididymis Dysfunction

Animal models of experimental varicocele have demonstrated structural and functional changes in the epididymis, revealing three critical sources of ROS, namely, metabolically active principal cells, endothelial cells from the capillary network around the epididymis caput, and the luminal fluid from the testis (28). These ROS accumulate primarily in the initial epididymal segment. However, the cells from all epididymal sections produce and release antioxidants in the epididymal fluid. Hypoxia and heat stress cause principal cells to generate excessive ROS, which, combined with impaired antioxidant production, result in oxidative damage to maturing sperm and epididymal cells (30).

### Varicocele and Sperm DNA Fragmentation

The WHO cut-off levels for basic SA parameters are poor predictors of natural pregnancy and ART success (13, 84-86). One reason is that routine SA does not include tests to assess sperm function, making it unable to diagnose alterations that could hamper embryo development and implantation (13, 84). Since varicocele is associated with OS, and one of the downstream effects of excessive ROS production is DNA damage, recent studies have focused on markers of DNA damage in assessing varicocele and sperm quality. These biomarkers include chromatin compaction, DNA methylation, and DNA fragmentation (87-90).

### Sperm DNA Fragmentation Tests

Several assays detect sperm DNA strand breaks. Some methods use enzymatic reactions to label the strand breaks (e.g., terminal deoxynucleotidyl transferase‐mediated dUTP‐biotin nick end labeling; TUNEL), while others use controlled DNA denaturation coupled with protein depletion to reveal the breaks (e.g., sperm chromatin structure assay [SCSA], sperm chromatin dispersion test [SCD], and the Comet assay) (91). A detailed analysis of assays' characteristics is beyond this article's scope and can be found elsewhere (91).

These tests measure the global sperm DNA fragmentation and provide information about sperm quality. Testing should be performed on neat semen samples after a standardized ejaculatory period of 2–3 days, as sperm DNA fragmentation levels increase with prolonged abstinence (92). Although each test detects DNA breaks using different strategies, thresholds of about 20% (by TUNEL, SCSA, SCD, and alkaline Comet) accurately discriminate between fertile and infertile men (93). Moreover, values greater than 20%–30% (by SCSA, alkaline Comet, and SCD) are optimal for classifying infertile couples into a statistical probability of prolonged time to achieve natural pregnancy, decreased likelihood of pregnancy by IUI, IVF, or ICSI and increased risk of miscarriage (91).

The sperm DNA fragmentation tests mentioned above are the most frequently used in clinical practice, and their results have a moderate-to-high correlation (94-96). Supporting these findings, a meta-analysis demonstrated an adverse effect of high sperm DNA fragmentation levels on clinical pregnancy rates after IVF/ICSI, irrespective of the measurement method (i.e., TUNEL, SCSA, SCD, and Comet) (97). Similarly, another meta-analysis demonstrated that the type of test used did not influence the positive effect of varicocelectomy on reducing sperm DNA fragmentation levels (23).

### Association Between Varicocele and Sperm DNA Fragmentation

High DNA fragmentation rates are frequently found in infertile men with varicocele (Table-2). Early studies revealed elevated sperm DNA fragmentation levels in infertile patients compared to fertile controls (44, 98). Smith and colleagues reported higher sperm DNA fragmentation levels in men with grade 2 or 3 varicocele than in healthy semen donors (26.1% ± 3.2% vs. 14.2% ± 1.2%, P<0.05), even when basic SA parameters were within the WHO reference ranges (98). The authors also demonstrated that a higher proportion of men with palpable varicocele and abnormal basic SA parameters had increased sperm DNA damage than men with varicocele and basic semen parameters within the reference ranges (58% vs. 49%, P-value not reported). This finding suggests that sperm DNA fragmentation levels increase as varicocele damage progresses.

**Table 2:** Characteristics of the studies assessing the effect of palpable varicocele on sperm DNA fragmentation

Author, year, (country)	SDF assay	Study groups	Main SDF results
Saleh et al., 2003 (USA) (44)	SCSA	16 fertile men without varicocele, 16 infertile men with palpable varicocele and 15 infertile men without varicocele	Control group: 15.0% (IQR: 10.0%, 22.0%); Infertile with varicocele: 25.0% (IQR: 20.0%, 35.0%) (P < 0.05 *versus* control); Infertile without varicocele: 20.0% (IQR: 13.0%, 28.0%)
Smith et al., 2005 (Chile) (98)	TUNEL and SCSA	25 healthy men without varicocele, 37 men with grade 2 and 3 varicocele and normal SA, 18 men with grade 2 or 3 varicocele and abnormal SA (fertility status not informed)	Control group: TUNEL 14.2% ± 1.2%; SCSA 7.1% ± 0.9%; Varicocele and normal SA group: TUNEL 26.1% ± 3.2%; SCSA 20.7% ± 4.0% (P < 0.05 *versus* control); Varicocele and abnormal SA group: TUNEL 32.2 ± 4.1%; SCSA 35.5% ± 9.0 % (P < 0.05 versus control)
Talebi et al., 2008 (Iran) (167)	SCSA	20 fertile men without varicocele and 20 infertile men with grade 2 or 3 varicocele	Control group: 17.3% ± 7.4%; Varicocele group: 60.5% ± 15.5% (P < 0.05 *versus* control)
Wu et al., 2009 (Taiwan) (168)	Comet	5 healthy men without varicocele and 15 men with grade 2 or 3 varicocele (fertility status not informed)	Control group: 4.5% ± 0.9%; Varicocele group: 8.4% ± 3.1% (P < 0.05 *versus* control)
Blumer et al., 2011 (Brazil) (165)	Comet	19 men without varicocele; 12 men with varicocele (fertility status not informed);	Class II sperm DNA fragmentation: Control: 51.3% ± 14.7 %; Men with varicocele: 59.4% ± 14.8% (P<0.05 *versus* control); There were no differences regarding the other three classes of sperm DNA fragmentation.
La Vignera et al., 2012 (Italy) (154)	TUNEL	30 fertile men without varicocele, 30 infertile men with grade 3 left varicocele and abnormal SA	Control group: 2.0% ± 1.0%; Varicocele group: 5.0% ± 3.0% (P < 0.05 *versus* control)
Li et al., 2012 (China) (169)	SCSA	19 healthy normozoospermic men and 19 infertile men with palpable varicocele and abnormal semen parameters	Control group: 17.4% ± 5.3%; Varicocele group: 28.4% ± 15.6% (P < 0.05 *versus* control)
Esteves et al., 2015 (Brazil) (101)	SCD	80 fertile donors and 98 infertile men with varicocele	Control group: 11.3% ± 5.5%; Varicocele group: 33.5% ± 18,3% (P < 0.05 *versus* control)
Alhathal et al., 2016 (Canada) (170)	SCSA	6 healthy normozoospermic men without varicocele, and 29 infertile men with palpable varicocele and abnormal semen parameters	Control group: 7.4% ± 5.0%; Varicocele group: 20.0% ± 10.6% (P < 0.05 *versus* control)
Ni et al., 2016 (China) (61)	SCSA	25 healthy normozoospermic men without varicocele, 19 infertile men with grade 1varicocele, 18 infertile men with grade 2 varicocele, and 14 infertile men with grade 3varicocele	Control group: 12.0% ± 7.9%; Varicocele grade 1 group: 23.6% ± 7.5% (P < 0.05 *versus* control); Varicocele grade 2 group: 27.7% ± 9.0% (P < 0.05 *versus control*); Varicocele grade 3 group: 30.0 % ± 8.3% (P < 0.05 *versus control*)
Abdelbaki et al., 2017 (Egypt) (42)	SCSA	20 fertile normozoospermic men without varicocele, and 60 infertile men with palpable varicocele and abnormal semen parameters	Control group: 7.6% ± 2.8%; Varicocele group: 29.9% ± 8.3% (P< 0.05 *versus* control)
Dieamant et al., 2017 (Brazil) (171)	TUNEL	2008 men without varicocele and 391 men with palpable varicocele (fertility status not informed)	Control group: 15.3% ± 8.5%; Varicocele group: 16.3% ± 8.8% (P < 0.05 *versus* control)
Santana et al., 2019 (Brazil) (107)	SCD	20 men without varicocele, and 19 men with varicocele (fertility status not informed)	Control group: 26.0% ± 10.0%; Varicocele group: 37.0% ± 20.0% (P = 0.09 *versus* control)
Lara-Cerrillo et al., 2020 (Spain) (172)	Comet	12 fertile men without varicocele and 20 infertile men with grades 2 or 3 varicocele	Control group: 45.0% ± 56.0%; Varicocele group: 53.0% ± 45.0% (P value not informed)
Tanaka et al., 2020 (Japan) (45)	SCSA	102 normozoospermic men without varicocele and 138 infertile men with palpable varicocele	Control group: 9.8% ± 10.3%; Varicocele group: 16.7% ± 12.1% (P < 0.05 *versus* control)
Ammar et al., 2021 (Tunisia) (51)	TUNEL	29 fertile men without varicocele; 11 infertile men with palpable varicocele and normal SA; 40 infertile men with varicocele and abnormal SA	Control group: 8.14% ± 6.86%; Varicocele and normal SA group: 60.87% ± 8.61% (P < 0.05 versus control); Varicocele and abnormal SA group: 69.88% ± 5.87% (P < 0.05 versus control)
Camargo et al., 2021 (Brazil) (166)	Comet	15 normozoospermic men without varicocele; 15 infertile men with grade 2 or 3 varicocele	Control group: 39.3% ± 11.69%; Varicocele group: 43.6% ± 11.9% (P = NS *versus* control)
Gil et al., 2021 (Poland) (46)	SCD	105 normozoospermic men without varicocele; 64 men with proven fertility; 71 infertile men with clinical varicocele; 95 infertile men without varicocele	Normozoospermic group: 13.3% ± 5.9%; Proven fertility group: 13.9% ± 7.1%; Varicocele group: 23.3% ± 11.9% (P < 0.05 versus normozoospermic and proven fertility); Infertile without varicocele: 19.4% ± 5.9% (P < 0.05 versus normozoospermic and proven fertility)
Jellad et al., 2021 (Tunisia) (106)	TUNEL	15 normozoospermic men without varicocele; 30 infertile men with palpable varicocele	Control group: 64.5% ± 17.7%; Varicocele group: 72.0% ± 15.3% (P < 0.05 *versus* control)
Jeremias et al., 2021 (Brazil) (99)	Comet	39 normozoospermic men without varicocele; 55 normozoospermic men with palpable varicocele	Control group: 39.3% ± 11.69%; Varicocele group: 43.6% ± 11.9% (P = NS *versus* control)

SDF: Sperm DNA Fragmentation; %SDF: sperm DNA fragmentation rate; TUNEL: terminal deoxynucleotidyl transferase‐mediated dUTP‐biotin nick end labeling; SCSA: sperm chromatin structure assay; SCD: sperm chromatin dispersion test; IQR: interquartile range; NS: not significant

Similarly, Ammar and colleagues reported that infertile men with palpable varicocele displayed increased sperm DNA fragmentation levels regardless of alterations in basic SA parameters; however, sperm DNA damage was greater in those with abnormal SA (51). Moreover, Jeremias and colleagues showed that men with varicocele can present with increased sperm DNA fragmentation even when basic semen analysis parameters are within the WHO reference ranges (99). Conversely, Ni and colleagues assessed sperm DNA fragmentation in infertile men with clinical varicocele and did not find increasing sperm DNA fragmentation rates after six months of observation compared to baseline, despite a worsening in the basic SA parameters (61). Interestingly, using an animal model, Carvalho and colleagues observed a negative progressive effect of varicocele on sperm DNA fragmentation (100).

A multicentric study involving 593 men with various causes of infertility found that sperm DNA fragmentation levels were the highest in men with varicocele (35.7% ± 18.3%) and in those with subclinical genital infection (41.7% ±17.6%) compared to a control group of fertile semen donors (11.3% ± 5.5%; P<0.05) (101). Moreover, two separate groups of sperm DNA breaks were identified: standard DNA fragmentation and degraded DNA fragmentation (DDS). Spermatozoa with standard fragmented DNA exhibited either the absence or presence of a small halo of chromatin dispersion around a compact nucleoid; in contrast, spermatozoa with degraded DNA showed a ghost-like morphology owing to massive single-strand and double-strand DNA breaks in addition to nuclear protein damage. In the study mentioned above, the proportion of sperm with degraded DNA was 8-fold higher in varicocele patients than in donors (54% ± 16% vs. 4.8% ± 7%; P<0.05). Interestingly, despite sperm with degraded DNA not being pathognomonic of varicocele, the index of sperm with degraded DNA reached an accuracy of 94% to discriminate between participants with and without varicocele (101).

Three systematic reviews have confirmed the link between varicocele and sperm DNA fragmentation. The first review by Zini and Dohle was a qualitative analysis of 16 case-control studies assessing sperm DNA fragmentation in fertile and infertile men with and without palpable varicocele (102). In four out of nine studies, sperm DNA fragmentation levels were higher in infertile men with clinical varicocele than in infertile counterparts without varicocele. Furthermore, men with clinical varicocele had worse SA parameters than infertile patients without varicocele. The remaining seven studies specifically included fertile men with clinical varicocele. In six of them, sperm DNA fragmentation rates were higher in men with clinical varicocele (and no history of infertility) than in fertile men or sperm donors without varicocele (102). This review indicates that varicocele not only increases sperm DNA fragmentation rates in men with infertility but also in those with "normal" fertility.

The second systematic review retrieved data from seven studies, including 240 patients with clinical varicocele and 176 controls without varicocele (103). The results revealed that sperm DNA fragmentation was higher in men with varicocele than in controls without varicocele (Mean difference: 9.84%; 95% CI: 9.19–10.49, P<0.05). However, the authors included studies with adolescents and pooled data irrespective of the assay used for sperm DNA fragmentation assessment.

The most recent meta-analysis compiled 12 case-control studies, including 875 participants with clinical and subclinical varicocele and 2377 men without varicocele (104). The authors reported a standard mean difference of 1.40% (95% CI: 0.83%-1.98%, P<0.05) between the groups. A subanalysis by type of sperm DNA fragmentation assay (TUNEL, Comet, and SCSA) revealed increased sperm DNA fragmentation in men with varicocele, irrespective of the assay utilized.

Only two studies looked into the proportion of men with varicocele who have increased sperm DNA fragmentation levels. Abdelaziz and colleagues analyzed a cohort of 54 infertile men with palpable varicocele and reported that 52% of them had sperm DNA fragmentation >30% (measured by TUNEL) (105). Moreover, another study found DNA fragmentation rates >30% in 21% of the infertile men with clinical varicocele, whereas only 1.5% of men with proven fertility demonstrated such high DNA damage, with an odd ratio of 16.8 (46).

Overall, current evidence indicates that men with palpable varicocele have increased sperm DNA fragmentation levels than men without varicocele. The effect is more evident in those men with abnormal basic SA parameters. Remarkably, the results are consistent and do not vary much with the type of test used. Nevertheless, the effect size fluctuated significantly, from 3% to 22%, possibly due to using different assays and the participant characteristics.

Some authors have investigated the influence of varicocele grade on sperm DNA fragmentation. Jellad and colleagues described that varicocele grade was positively associated with sperm DNA fragmentation (15.2% ± 1.9% in grade 3 vs. 12.9% ± 3.5% in grade 2, P<0.05) (106). Similarly, young men (aged 16 to 26) with grade 3 varicocele demonstrated increased sperm DNA fragmentation levels compared to those with grades 1 and 2 (71). Moreover, the study by Ni and colleagues assessed sperm DNA fragmentation in men with palpable varicocele and found numerically higher levels of DNA damage as varicocele grade increased (23.6% ± 7.5% in grade 1, 27.7% ± 9.0% in grade 2, and 30.0% ± 8.3 in grade 3; P value not reported) (61). In contrast, Santana and colleagues reported no differences in sperm DNA fragmentation levels between men with grade 2 and 3 varicoceles (41% ± 24% vs. 34% ± 13%, P=0.99) were reported (107). The only study examining the influence of laterality on sperm DNA damage reported that sperm DNA fragmentation levels were higher in men with bilateral varicocele than in those with unilateral varicocele (16.4% ± 10.1% vs.12.0% ± 8.8%, P < 0.05) (108). Given the limited data available, further research into this matter is warranted.

There is even less published data about the effect of subclinical varicocele on sperm DNA fragmentation levels (109). García-Peiró and colleagues, using SCD, demonstrated that men with subclinical varicocele have increased sperm DNA fragmentation levels compared to fertile donors (37.5% vs.12.0%, P value not reported) (110). In contrast, Ni and colleagues reported no differences between infertile men with subclinical varicocele and fertile men without varicocele (14.9% ± 5.1% vs.12.0% ± 7.9, by SCSA), even though men with subclinical varicocele had significantly lower basic SA parameters than controls (P<0.05) (61). Additionally, the authors demonstrated that patients with subclinical varicocele had no deterioration of sperm DNA fragmentation levels over a 6-month follow-up (61).

Evidence of the association between varicocele and elevated sperm DNA fragmentation has been increasing steadily (111, 112). Similarly, other markers of sperm function, including epididymal neutral α-glucosidase and sperm PLCζ levels, are also reduced in men with high SDF and grade II or III varicocele (111).

### Impact of Varicocele Repair on Oxidative Stress and Sperm DNA Fragmentation

#### Varicocelectomy Techniques and Rationale

Varicocele repair is typically recommended for infertile men with a palpable disease and abnormal basic SA parameters (113-115) since improvements in basic semen parameters and pregnancy outcomes after varicocelectomy are consistently observed in these individuals (12, 116). Conversely, varicocele repair is not routinely recommended for males with subclinical varicocele due to the contradictory evidence regarding the benefit in this population (117-119).

Surgical repair has been the standard treatment for infertile men with varicocele since Celsus, in the first century A.D., performed the first documented varicocelectomy (120). The main goal of varicocele repair is the occlusion of varicose veins of the pampiniform plexus and their collateral drainage via the external spermatic and cremasteric veins while preserving testicular arteries, lymphatics, and nerves (121, 122). Several techniques have been applied, including open surgical methods (with or without microsurgery), laparoscopy, and embolization. In the open technique, ligation of the veins is performed via retroperitoneal, inguinal, or subinguinal incisions (123). With laparoscopy, the spermatic veins are occluded a few centimeters from the internal inguinal orifice (124). Radiological embolization is carried out via femoral or jugular veins, and the interruption of venous flow through the internal spermatic and collateral veins is achieved using embolic agents (125).

**Figure 2:**
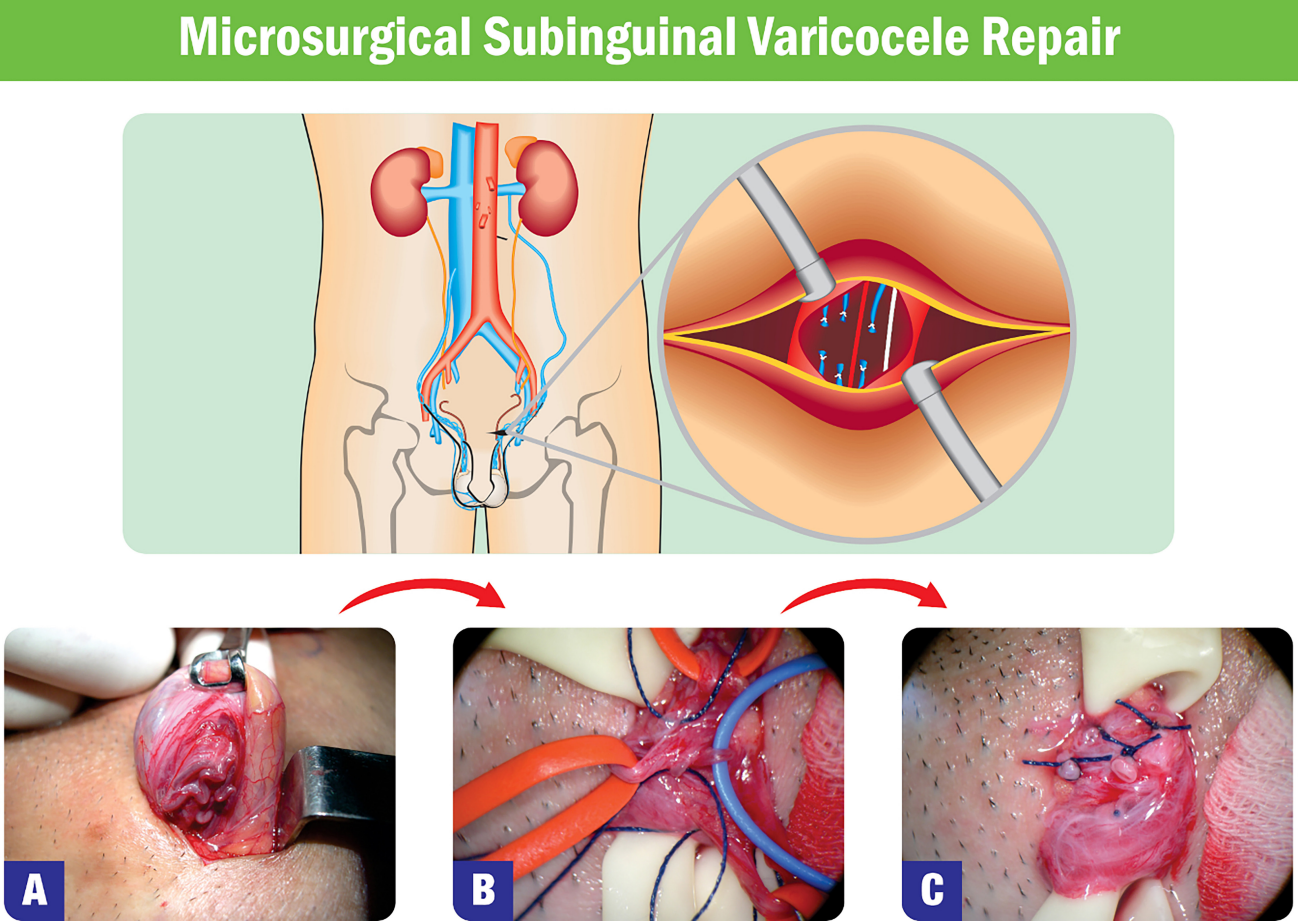
Microsurgical Varicocele Repair.

The gold-standard treatment is microsurgical varicocelectomy (MV) (126). Its main surgical steps are illustrated in Figure-2. The improvement rate of basic SA parameters varies from 64 to 81% after MV, and the likelihood of improvement positively correlates with varicocele grade (15, 127-129). Total motile sperm count increases after varicocele repair, which may allow couples needing ICSI to use less invasive assisted conception modalities (e.g., IUI) or attempt natural pregnancy (130). Moreover, the latest Cochrane meta-analysis demonstrated that varicocelectomy increases natural pregnancy rates compared with delayed or no treatment in infertile men with palpable varicocele and abnormal basic SA parameters (RR 1.94, 95% CI 1.23–3.05, P<0.05, seven randomized controlled trials; 693 participants) (131). The authors reported that, on average, six patients would have to undergo varicocelectomy for one additional couple to achieve a natural pregnancy.

A ~2-cm transversal skin incision is made below the external inguinal ring (upper). The muscle layers and the inguinal canal are not violated. The lower panels show intraoperative photographs of the spermatic cord structures. The spermatic cord is exteriorized, and the cremasteric veins are identified and ligated (A). In panel B, the spermatic cord is dissected to allow the identification of the testicular artery (blue vessel loop), dilated varicose veins (red vessel loops), and lymphatics (blue cotton sutures). The testicular artery and lymphatic channels are preserved, whereas the dilated veins are ligated with nonabsorbable sutures and transected (C).

There is a broad variation in the natural pregnancy rates after MV, ranging from 29% to 60% during the first 12 months after the procedure (127, 132). Factors such as female infertility, baseline semen parameters, varicocele severity, and other associated male comorbidities impact the reproductive outcomes of MV and contribute to this wide variation. Varicocele repair also increases pregnancy rates by ICSI compared to couples whose male partners did not undergo treatment (clinical pregnancy: OR 1.59, 95% CI 1.19–2.12, P<0.05), four observational studies, 852 participants; live birth: OR 2.17, 95% CI 1.55–3.06, P<0.05, three observational studies, 622 participants) (20). Based on these findings, five patients, on average, would have to receive varicocele repair before ICSI (versus no treatment) for one additional couple to achieve a pregnancy.

Furthermore, microsurgical varicocelectomy increases intratesticular testosterone production, an essential process for normal spermatogenesis (133). A meta-analysis evaluating the impact of varicocele repair on the testosterone levels of hypogonadal men (i.e., having serum total testosterone levels below 300 ng/dL) reported an increase of 123 ng/dL in the total testosterone levels compared to the preoperative levels (P<0.05) (134).

### Impact of varicocelectomy on Oxidative Stress

Since varicocele is associated with excessive levels of ROS in the semen, some authors have evaluated the role of varicocele repair in alleviating seminal OS (Table-3). Dada and colleagues demonstrated a decrease in the ROS levels measured by the chemiluminescence method using luminol as a probe in 11 men with clinical varicocele one month after varicocelectomy (preoperative: 142,897.704 RLU per 20 million sperm/min vs. postoperative: 10,776.736 RLU per 20 million sperm/min; P<0.001) (135). The authors also reported a further decrease in ROS levels in men who returned for follow-up after six months of varicocelectomy (135). Similarly, Abdelbaki and colleagues reported reduced ROS levels measured by chemiluminescence and increased TAC in a cohort of 55 men who underwent varicocele repair (42). Furthermore, using seminal MDA measurement to assess ROS levels, Ni and colleagues demonstrated an improvement of OS in infertile men with all three grades of palpable varicocele at 3 and 6 months after MV (61). However, the authors did not find differences in seminal MDA levels between couples who achieved natural pregnancy after MV and those who did not. Additionally, Tavalaee and colleagues used 20, 70-dichlorodihydrofluorescein diacetate (DCFH) to evaluate intracellular ROS levels and demonstrated that the mean percentages of DCFH-positive spermatozoa decreased postoperatively (from 47.6% to 36.6%; P=0.03) (136). Also, applying DCFH to evaluate OS, Barekat and colleagues reported an increase in the percentage of ROS-negative sperm (77.2% ± 7.5% vs. 92.3% ± 2.6%, P<0.05) 3 months after MV (137). Moreover, Abbasi and colleagues assessed sperm lipid peroxidation as an OS marker and described improvement after MV (36.22% vs. 24.04%; P=0.009) (138). Measuring the static oxidation-reduction potential (sORP) preoperatively and three months postoperatively, Kavoussi and colleagues found decreased sORP in infertile men with palpable varicocele who underwent MV (preoperative: 4.73 mV/106 sperm/ml vs. postoperative: 2.03 mV/106 sperm/ml, P<0.001) (139). Notably, the authors also described improved sperm DNA fragmentation levels after surgery; however, there was no correlation between sORP and sperm DNA damage.

**Table 3:** Characteristics of the studies assessing the effects of varicocelectomy on oxidative stress

Author, year, (country)	ROS assay	Study groups	Main results
Mancini et al., 2004 (Italy) (140)	Seminal TAC by Colorimetric assay kit	25 infertile men with varicocele 14 infertile men who underwent varicocelectomy 10-24 months before	**TAC:** Preoperative: 106.6 ± 8.9 seconds; Postoperative: 105.8 ± 8.7 seconds (P NS *versus* preoperative)
Sakamoto et al., 2008 (Japan) (38)	Seminal NO levels; Seminal 8-OHdG levels; Seminal SOD activity	Not reported	**NO levels:** Preoperative: 17.1 ± 9.1μmol/L; 6 months postoperative: 7.5 ± 4.5μmol/L (P<0.05 versus preoperative) **8-OHdG levels:** Preoperative: 10.3 ± 4.7 μmol/L; 6 months postoperative: 6.2 ± 2.5 μmol/L (P<0.05 *versus* preoperative) **SOD activity:** Preoperative: 85.8 ± 5.8%; 6 months postoperative: 78.1 ± 8.1% (P<0.05 *versus* preoperative)
Dada et al., 2010 (India) (135)	Seminal ROS by chemiluminescence with luminol	11 infertile men with palpable varicocele	**ROS levels:** Preoperative: 142,897.704 RLU per 20 million sperm/min 1 month postoperative: 10,776.736 RLU per 20 million sperm/min (P < 0.05 *versus* preoperative); 3 months postoperative: 6,456.249 RLU per 20 million sperm/min (P < 0.05 *versus* preoperative)
Baker et al., 2013 (USA)	Seminal ROS by chemiluminescence with luminol; Seminal TAC by Colorimetric assay kit	24 infertile men with palpable varicocele	**ROS levels:** Preoperative: 1185.1 RLU/sec/10^6^; 3 months postoperative: 2710.911851 RLU/sec/10^6^ (P not reported) **TAC:** Preoperative: 2292 µM Trolox; 3 months postoperative: 1885 mM Trolox (P < 0.05 *versus* preoperative); % patients with TAC above normal: Preoperative: 86%; 3 months postoperative: 71% (P value not reported)
Tavalaee et al., 2015 (Iran) (136)	Seminal OS by DCFH-DA staining	23 infertile men with varicocele grade II and III	**DCFH-DA negative spermatozoa:** Preoperative: 37.2% ± 3.6 %; 3 months postoperative: 61.3% ± 5.3 % (P < 0.05 *versus* preoperative)
Barekat et al., 2016 (Iran) (137)	Seminal OS by DCFH-DA staining	20 infertile men with varicocele grade II and III	**DCFH-DA positive spermatozoa:** Preoperative: 47.6% ± 6.6 %; 3 months postoperative: 36.6% ± 3.8 % (P < 0.05 *versus* preoperative)
Abdelbaki et al., 2017 (Egypt) (42)	Seminal ROS by chemiluminescence with luminol; Seminal TAC by Colorimetric assay kit	55 infertile men with palpable varicocele	**ROS levels:** Preoperative: 4.49 ± 0.9 log[ROS+1]; 3 months postoperative: 3.27 ± 1.3 log[ROS+1] (P < 0.05 *versus* preoperative) **TAC:** Preoperative:1.01 ± 0.4 mM Trolox; 3 months postoperative: 2.05 ± 0.5 mM Trolox (P < 0.05 *versus* preoperative)
Ni et al., 2016 (China) (61)	Seminal MDA by thiobarbituric acid reaction	19 infertile men with grade I varicocele; 18 infertile men with grade II varicocele; 14 infertile men with grade III varicocele	**MDA levels:** Preoperative varicocele grade I group: 12.18 ± 4.86 nmol/mL; 3 months postoperative varicocele grade I group: 9.88 ± 3.98 nmol/mL (P NS *versus* preoperative); 6 months postoperative varicocele grade I group: 8.76 ± 2.73 nmol/mL (P < 0.05 *versus* preoperative); Varicocele grade II group: 14.12 ± 5.42 nmol/mL; 3 months postoperative varicocele grade II group: 9.22 ±3.75 nmol/mL (P < 0.05 *versus* preoperative) 6 months postoperative varicocele grade II group: 9.71 ± 2.83 nmol/mL (P < 0.05 *versus* preoperative); Varicocele grade III group: 15.86 ± 6.78 nmol/mL; 3 months postoperative varicocele grade II group: 11.38 ± 3.94 nmol/mL (P < 0.05 *versus* preoperative); 6 months postoperative varicocele grade III group: 9.50 ± 3.28 nmol/mL (P < 0.05 *versus* preoperative)
Abbasi et al., 2020 (Iran) (138)	Lipid peroxidation by the BODIPY probe	22 infertile men with varicocele grade II and III	**BODIPY-positive spermatozoa:** Preoperative: 36.22% ± 3.38 %; 80 days postoperative: 24.04% ± 1.80 % (P < 0.05 *versus* preoperative)
Kavoussi et al., 2022 (USA) (139)	ORP by MiOXSYS System	49 infertile men with palpable varicocele	**ORP:** Preoperative:4.73 mV/10^6^ sperm/mL; 3 months postoperative: 2.03 mV/106 sperm/mL (P < 0.05 *versus* preoperative)

Cat :catalase activity; DCFH-DA: 20, 70-dichlorodihydrofluorescein diacetate; GPx: glutathione peroxidase activity; H2O2: hydrogen peroxide; IQR: interquartile range; MDA: malondialdehyde; NO: Nitric oxide; NS: not significant; ORP: oxidation-reduction potential; ROS: Reactive oxygen species; SA: semen analysis; SOD: superoxide dismutase activity; TAC: total antioxidant capacity;

Conversely, Mancini and colleagues, comparing TAC values between 25 infertile men with varicocele and 14 infertile men who had undergone MV 10-24 months before (140), did not find a difference between the two groups (106.6 ± 8.9 seconds vs. 105.8 ± 8.7 seconds). Moreover, while reporting a decrease in TAC from 2292 uM preoperatively to 1885 uM postoperatively (P=0.03), Baker and colleagues noticed that most participants persisted with TAC above the reference level (141). Additionally, the authors did not find a statistically significant difference in ROS or ROS-TAC scores after the procedure. The limited evidence points towards a beneficial effect of varicocelectomy in reducing OS in semen samples of infertile men.

### Sperm DNA Fragmentation Levels After Varicocelectomy and Outcomes

Given the vital link between varicocele and sperm DNA fragmentation, the role of varicocele repair in improving sperm DNA has been under scrutiny (63). To date, four meta-analyses have been reported on this topic, and their findings will be summarized in this section (Table-4).

**Table 4:** Characteristics of the meta-analyses assessing the effects of varicocelectomy on sperm DNA fragmentation

Author, year, (country)	Population	Type of Included Studies	SDF assay	Varicocelectomy technique	Number of studies and participants	Decrease %SDF after varicocelectomy	Limitations
Wang et al., 2012 (China) (103)	Infertile men with palpable varicocele and abnormal SA	Retrospective and prospective cohort	SCSA, TUNEL and Comet	Open non-microsurgical and open microsurgical	6 studies; 177 participants	WMD -3.37%; 95% CI: -4.09 to -2.65, P<0.05	One study included men using antioxidants, and another study included adolescents; Data were pooled irrespective of SDF assay type; Pregnancy and live birth rates not evaluated
Qiu et al., 2020 (China) (144)	Men with varicocele	Prospective cohort and case-control	SCSA, SCD, TUNEL, Comet, and AOT	Open non-microsurgical and open microsurgical	11 studies; 394 participants	WMD -5.79%; 95% CI -7.39 to -4.19, P<0.05	One study included fertile men, another included men with subclinical varicocele; one study included adolescents, and another trial assessed SDF by a sperm chromatin protamination test; data was pooled irrespective of SDF assay type; pregnancy and live birth rates not evaluated
Birowo et al., 2020 (Indonesia) (147)	Infertile men with palpable varicocele	Prospective cohort	SCSA and TUNEL	Open non-microsurgical and open microsurgical	7 studies; 289 participants	WMD -6.86%; 95% CI -10.04 to -3.69, P<0.05	Low number of studies and participants; data was pooled irrespective of SDF assay type; pregnancy and live birth rates not evaluated
Lira Neto et al., 2020 (Brazil) (23)	Infertile men with palpable varicocele	Retrospective and prospective cohort	SCSA, SCD, TUNEL and Comet	Open non-microsurgical, open microsurgical, and laparoscopic	19 studies; 1070 participants	WMD -7.23%; 95% CI -8.86; -5.59; P<0.05	Pregnancy and live birth rates not assessed

AOT: Acridine orange test; SDF: Sperm DNA Fragmentation; %SDF: sperm DNA fragmentation rate; TUNEL: terminal deoxynucleotidyl transferase‐mediated dUTP‐biotin nick end labeling; SCSA: sperm chromatin structure assay; SCD: sperm chromatin dispersion test; WMD: Weight Mean Difference

The first meta-analysis was published in 2012 by Wang and colleagues. The analysis included data from six studies involving 177 men with clinical varicocele (103). The authors reported a statistically significant reduction (weighted mean difference [WMD] of -3.4%; 95% CI: -4.1 to -2.5, P<0.05) in the sperm DNA fragmentation rates after varicocelectomy. However, these authors included one study of men using antioxidants (142) and another on adolescents (143). Additionally, they pooled the data irrespective of the type of assay used for sperm DNA fragmentation measurement.

In 2020, Qiu and colleagues performed a new meta-analysis including 394 participants from 11 studies and confirmed the findings of the previous study by Wang and colleagues. However, in their study, a larger effect size of varicocelectomy on sperm DNA fragmentation rates was found (WMD -5.79%; 95% CI -7.39 to -4.19, P<0.05) (144). The limitations of this study were the inclusion of one study with men who had varicocelectomy for reasons other than infertility (145), a study involving men with subclinical varicocele (110), another including adolescents (143), and a trial assessing sperm DNA fragmentation by the sperm chromatin protamination test (146), which is not optimal for detecting DNA strand breaks. Moreover, this study also pooled the data irrespective of the type of assay used for sperm DNA fragmentation measurement.

The meta-analysis by Birowo and colleagues, also published in 2020, analyzed seven prospective studies, including in total 289 infertile men with palpable varicocele, and found a reduction in sperm DNA fragmentation rates after varicocelectomy (WMD -6.9%; 95% CI -10.0% to -3.7%, P<0.05) (147). This study included few trials and participants and examined only the SCSA and TUNEL for sperm DNA fragmentation assessment. Moreover, a subanalysis by the type of sperm DNA fragmentation assay was not carried out.

In the most recent systematic review and meta-analysis, our group compiled data from 19 studies involving 1070 infertile men with palpable varicocele. In our study, varicocelectomy reduced postoperative sperm DNA fragmentation rates (all sperm DNA fragmentation assays combined; WMD -7.2%; 95% CI -8.9%; -5.6%; P<0.05) with a moderate effect size (Cohen's d=0.68; 95% CI: [WMD] 0.77-0.60) (23). When the studies were categorized by the type of sperm DNA fragmentation assay (TUNEL, SCSA, SCD, and alkaline Comet), the reduction in sperm DNA fragmentation levels remained significant, without major variation among assays. These findings align with studies demonstrating a moderate-to-high correlation between the assays used to measure sperm DNA fragmentation (94-96, 148). Furthermore, they corroborate recent data indicating a substantial intraindividual agreement in sperm DNA fragmentation rates evaluated in two ejaculates from the same subjects within a 3-month interval (149).

In the study mentioned above, we have also evaluated the influence of the surgical technique on the improvement of sperm DNA fragmentation and found a similar effect size for microsurgical (WMD -7.2%, 95% CI -8.9%, -5.4%; P<0.05) and open non-microsurgical techniques (WMD -7.1%, 95% CI -12.7%, -1.5%; P<0.05). Corroborating this finding, a comparative review of different approaches for varicocele repair revealed that open techniques, mainly microsurgery, yielded more significant improvements in semen parameters and pregnancy rates than other techniques (123).

**Figure 3:**
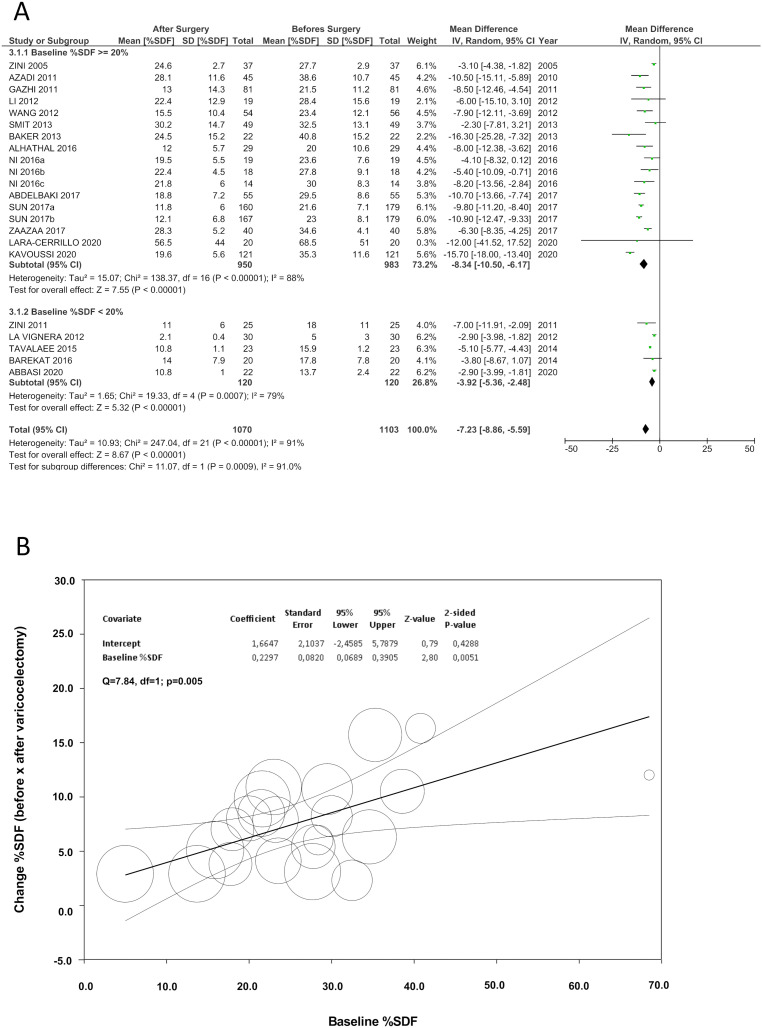
Varicocele Repair on Sperm DNA Fragmentation.

Furthermore, in subanalysis by baseline sperm DNA fragmentation levels, we demonstrated that men with preoperative levels >20% had a more significant reduction of sperm DNA fragmentation compared to those with levels <20% (all sperm DNA fragmentation assays combined; WMD -8.3% vs. -3.9%, P<0.05). Furthermore, we conducted a meta-regression analysis revealing that sperm DNA fragmentation improved postoperatively as a function of preoperative sperm DNA fragmentation levels (Coefficient: 0.23; 95% CI: 0.07-0.39; P<0.05) (Figure-3). These findings suggest that men with high sperm DNA fragmentation levels at baseline benefit the most from varicocele repair, similar to the recommendations of varicocelectomy regarding basic semen analysis parameters (12).

(A) Forest plot showing the Weighted Mean Difference (WMD) for sperm DNA fragmentation (SDF) rates after varicocelectomy (versus before) according to baseline (preoperative) SDF levels. CI = confidence interval; IV = inverse variance; (B) Meta-regression showing the influence of baseline SDF levels (moderator) on %SDF change after varicocelectomy. The size of the circles indicates each study's sample size. The solid line represents linear predictions for the %SDF change after varicocelectomy as a function of the mean absolute increase in preoperative SDF levels. The curved lines indicate the 95% confidence interval around the regression line (42, 61, 136-138, 141, 151-157, 169, 170, 172-175). Reprinted with permission from Elsevier; Lira Neto et al. Effect of varicocelectomy on sperm deoxyribonucleic acid fragmentation rates in infertile men with clinical varicocele: a systematic review and meta-analysis. Fertil Steril. 2021 Sep;116(3):696-712.

Concerning the improvement of sperm DNA fragmentation to levels lower than the threshold of 30%, Werthman and colleagues studied 11 infertile men with palpable varicocele, abnormal basic SA, and baseline SDF>30%. The authors reported that 64% of the participants reached SDF levels <30% 3 to 6 months after varicocelectomy (150). Similarly, Ghazi and colleagues found that 88% of men with preoperative sperm DNA fragmentation >30% improved to levels <30% following MV (151).

The influence of varicocele grade on the outcomes of varicocelectomy has been highlighted by a recent meta-analysis that showed a greater improvement in basic SA parameters in men with grade 2 and 3 varicocele (114). Despite the association between sperm DNA fragmentation improvement in all varicocele grades mentioned in our study (23), we could not perform a subanalysis by grade due to the small number of studies that provided such data (61, 152-154).

*Subclinical Varicocele*. Only two studies have investigated the effect of repairing subclinical varicoceles on sperm DNA fragmentation levels. The study by Garcia-Peiró and colleagues included infertile men with subclinical varicocele diagnosed by scrotal Doppler ultrasonography and found no difference in the sperm DNA fragmentation levels between the participants who underwent varicocelectomy and those who did not (31.4% vs. 28.9%, by TUNEL) (110). Furthermore, employing SCSA to measure sperm DNA fragmentation, Sun and colleagues evaluated 358 infertile men with left clinical and right subclinical varicocele, randomized to undergo bilateral (n = 179) or unilateral (n = 179) microsurgical subinguinal varicocelectomy (155). The authors reported more significant improvement in basic semen analysis parameters and higher natural pregnancy rates in the bilateral varicocele repair group compared with the unilateral varicocele group. However, sperm DNA fragmentation levels were not statistically different among the groups both preoperatively (21.6% ± 7.1% vs. 23.0% ± 8.1%) and postoperatively (11.8% ± 6.0% vs. 12.1% ± 6.8%) (155).

The timing for sperm DNA fragmentation retesting after varicocelectomy has also been studied. Most authors recommend a follow-up test between 3 to 6 months after the procedure, similar to the recommendation regarding basic SA parameters. This suggestion is based on the duration of spermatogenesis in humans, which is approximately 72 days (11). Thus, waiting more than 90 days ensures that at least one wave of spermatogenesis has progressed under the procedure's benefit. Some studies have demonstrated a progressive decline in sperm DNA fragmentation levels with increasing follow-up time after varicocelectomy (105). In contrast, others found consistently lower sperm DNA fragmentation levels in the postoperative period (e.g., three months), without further significant improvement over time (61, 156).

The association between the improvement of sperm DNA fragmentation and reproductive outcomes has been the objective of few studies. Smit and colleagues, studying infertile men with palpable varicocele and oligozoospermia, found lower postoperative sperm DNA fragmentation levels in couples that conceived naturally or with ART exhibited compared to those who did not (26.6% ± 13.7% vs. 37.3% ± 13.9%, P<0.05) (157). Similarly, Ni and colleagues demonstrated that infertile men with palpable varicocele and abnormal semen analysis who achieved pregnancy naturally six months after varicocelectomy had decreased sperm DNA fragmentation rates compared to preoperative values (17.6% ± 3.4% vs. 26.8% ± 8.6%, P<0.05) and non-pregnant patients (17.6 ± 3.4% vs. 22.3 ± 5.4%; P<0.05) (61). Likewise, Wang and colleagues found that the mean postoperative sperm DNA fragmentation rate in infertile men with clinical varicocele and elevated sperm DNA fragmentation levels who underwent varicocele repair and fathered a child was lower than in those who did not conceive (13.9% ± 9.7% vs. 20.1% ± 10.3%, P<0.05), (152). Furthermore, in a prospective study including 75 infertile men with palpable varicocele and abnormal SA parameters, Mohammed and colleagues reported that couples that achieved natural pregnancy at 1-year follow-up after the procedure had significantly lower sperm DNA fragmentation levels than those who did not (16.4% ± 6.4% vs.24.2 ± 4.1%, P<0.05) (158). In contrast, in a retrospective study including 24 infertile men with palpable varicocele, no difference in sperm DNA fragmentation levels was found between pregnant and non-pregnant couples after MV (22.2% ± 14.4% vs. 25.7% ± 14.5%, P=0.6), despite a significant decrease in the mean sperm DNA fragmentation rates after the operation (preoperative: 40.8% vs postoperative: 24.5%; P<0.05) (141).

The studies summarized above indicate that varicocele repair in infertile men with palpable varicocele reduces sperm DNA fragmentation levels. Furthermore, sperm DNA integrity improvement after the intervention seems to enhance the chance of natural conception. However, data substantiating the latter statement is still limited and overwhelmingly based on observational studies.

### Practice change: Updated Varicocelectomy Indications

In most practice guidelines from professional societies, varicocele repair is only recommended for infertile men with palpable varicocele and abnormal basic semen parameters (i.e., sperm concentration, sperm motility, or sperm morphology) (12). However, basic semen analysis parameters lack accuracy in assessing the male fertility potential (13, 84-86, 159). Moreover, recent evidence referenced in this review highlights the critical relationship among varicocele, OS, and sperm DNA fragmentation, as well as their negative effect on male fertility. Therefore, this topic has been revisited recently, and new guidelines have already suggested that elevated sperm DNA fragmentation levels should be considered an indication of varicocelectomy repair in infertile males with palpable varicocele, even for patients with basic SA parameters within the WHO normal ranges.

For instance, the latest European Urology Association (EAU) male infertility guideline includes a strong recommendation for sperm DNA fragmentation assessment in men with otherwise unexplained infertility or who have reported failed ART, including recurrent pregnancy loss or failure of embryo development and implantation (116). The same document goes further, including a weak recommendation for varicocelectomy in men with elevated sperm DNA fragmentation levels in the same scenarios (i.e., unexplained infertility, recurrent pregnancy loss, failure of embryo development or implantation). In addition, the guidelines highlight that OS has been recognized as a cause of male infertility, albeit stating that seminal ROS testing cannot be recommended in routine clinical practice until its diagnostic utility is validated by further studies (116).

The Sperm DNA Fragmentation Study Group (SFRAG) issued a guideline summarizing the evidence concerning the impact of sperm DNA fragmentation in different clinical settings, such as varicocele, unexplained infertility, idiopathic infertility, recurrent pregnancy loss, intrauterine insemination, in vitro fertilization/intracytoplasmic sperm injection, fertility counseling for men with infertility risk factors, and sperm cryopreservation (24). Regarding varicocele, the SFRAG guideline states that assessing sperm DNA fragmentation levels in infertile men is helpful when deciding about treatment options, especially in men with low-grade varicocele or in those with normal or borderline basic SA parameters. Furthermore, it highlights that determining postoperative sperm DNA fragmentation levels helps guide further treatments (24). The SFRAG guideline also provides helpful recommendations for the decision-making process when dealing with infertile men with varicocele, even in cases where varicocelectomy is not warranted by itself according to the traditional indication, i.e., when basic semen parameters are within the reference ranges. For instance, it states that sperm DNA fragmentation testing might also be helpful for infertile men with palpable varicocele who are candidates for ART. In these cases, varicocelectomy could be considered before ART for patients with elevated sperm DNA fragmentation to improve reproductive outcomes (160). Importantly, as reasoned by researchers in recent articles, only through a comprehensive andrological evaluation, including a detailed medical and reproductive history and physical examination, and additional investigations such as sperm DNA fragmentation testing, will correctable conditions such as varicocele be detected and optimally treated, allowing the couple to achieve the best reproductive outcomes possible potentially (13, 84, 161).

### Knowledge Gaps and Future Research

Current literature indicates a strong link between varicocele-related infertility and OS. However, further data is needed from prospective studies, including fertile controls and fertile and infertile men with varicocele, with large sample sizes from various institutions and countries, to better describe the prevalence and natural history of OS among men with varicocele. In addition, standardization of the methods used for OS assessment, as well as subanalyses by varicocele grade and laterality, should be performed in these studies.

Regarding the impact of varicocelectomy on seminal ROS levels, there is level 2 evidence indicating an improvement of OS markers in infertile men with varicocele. However, due to the small number of studies and the lack of standardization of the different methods used to assess OS, further prospective studies with larger sample sizes and simultaneously applying direct and indirect techniques to measure ROS are needed to produce stronger evidence. Additionally, subanalyses by varicocele grade, laterality, type of ROS assay, and baseline ROS levels should be carried out. More importantly, the relationship between the improvement of OS after varicocele repair and reproductive outcomes must be evaluated.

Level 1 evidence concerning the negative association between varicocele and sperm DNA fragmentation and the positive effect of varicocele repair on sperm chromatin integrity already exists. Nevertheless, some points remain to be elucidated. The exact prevalence and natural history of elevated sperm DNA fragmentation among varicocele patients are still unclear. Similarly, data concerning the influence of varicocele grade and laterality on preoperative and postoperative sperm DNA fragmentation levels is limited. Thus, larger and multicentric cohort studies and clinical trials with subgroup analyses by varicocele grade and laterality are needed.

Future research is also warranted to clarify whether varicocelectomy can also improve sperm DNA fragmentation in men with basic SA parameters within the WHO reference ranges, as well as the proportion of patients with high baseline sperm DNA fragmentation levels that reach normal levels after varicocelectomy (162). Clinical trials including this population should be performed, ideally including a group of infertile men with varicocele, abnormal basic SA parameters, and high sperm DNA fragmentation to compare outcomes. Moreover, further studies should assess sperm DNA fragmentation levels at different time intervals after varicocele repair and their relationship with pregnancy outcomes in both natural and ART scenarios. Clinical trials with serial postoperative measurements of sperm DNA fragmentation and a follow-up of at least 12 months are needed. Preoperative sperm DNA fragmentation levels may also be included in nomograms created to predict the reproductive outcomes of varicocele repair at the individual level.

Lastly, there are knowledge gaps concerning the specific pathways by which varicocele causes OS and sperm DNA fragmentation and how varicocelectomy improves sperm chromatin integrity and decreases ROS production. Studies using ‘omics' techniques may illuminate the relevant metabolic pathways (48, 163, 164). Table-5 summarizes the main knowledge gaps and the further research needed, as discussed above.

**Table 5:** Main knowledge gaps regarding varicocele, oxidative stress, and sperm DNA fragmentation

Knowledge gaps	Suggested studies
Mechanisms by which varicocele causes OS.	Proteomics and Metabolomics studies in men with and without varicocele.
Impact of varicocele laterality on OS.	Cross-sectional studies in infertile men with unilateral *versus* bilateral varicocele.
Impact of subclinical varicocele on OS.	Cross-sectional studies in infertile men with subclinical *versus* palpable varicocele.
Definition of cut-off levels for the different OS markers for men with varicocele	Cross-sectional studies including healthy fertile normozoospermic men (controls), fertile men with varicocele, and infertile men with varicocele, using several markers of OS (Total ROS, MDA, 8-OHdG, and TAC) simultaneously, providing ROC curve analysis for each marker.
Impact of time on varicocele-induced OS.	Prospective cohort studies with fertile and infertile men with varicocele measuring OS markers in serial time points.
Impact of varicocelectomy on varicocele-induced OS	Prospective cohort studies including infertile men measuring various OS markers before and after varicocele repair, including subanalyses by varicocele grade, laterality, baseline OS marker levels, and surgical technique. Measuring various OS markers in serial time points is also recommended.
Impact of time on varicocele-induced sperm DNA fragmentation.	Prospective cohort studies with fertile and infertile men measuring sperm DNA fragmentation in serial time points.
Proportion of men with varicocele and increased sperm DNA fragmentation levels	Cross-sectional studies including healthy, fertile normozoospermic men (controls), fertile men with varicocele, and infertile men with varicocele. Cut-off levels should be defined for each assay based on the literature.
Impact of varicocele grade on sperm DNA fragmentation.	Cross-sectional studies in infertile men grouped by varicocele grade (including subclinical).
Impact of varicocele laterality on sperm DNA fragmentation.	Case-control studies in infertile men with unilateral *versus* bilateral varicocele.
Impact of varicocelectomy on varicocele-induced sperm DNA fragmentation	Prospective cohort studies including infertile men with increased sperm DNA fragmentation levels undergoing varicocele repair. Subanalyses by varicocele grade, laterality, and surgical technique should be performed. Measuring sperm DNA fragmentation in serial time points is also recommended.
Association between OS and sperm DNA fragmentation levels in men with varicocele	Cross-sectional studies including healthy fertile normozoospermic men (controls), fertile men with varicocele, and infertile men with varicocele, and measuring simultaneously several OS markers as well as sperm DNA fragmentation
Association between the improvement of OS and sperm DNA fragmentation levels after varicocele repair.	Prospective cohort studies including infertile men with increased OS markers and sperm DNA fragmentation levels undergoing varicocele repair. Several OS markers and sperm DNA fragmentation should be measured simultaneously and in serial time points.
Association between the improvement of OS and sperm DNA fragmentation levels after varicocele repair with natural pregnancy outcomes.	Prospective cohort studies including infertile men with increased OS markers and sperm DNA fragmentation levels undergoing varicocele repair. Participants should be followed up for at least 12 months after surgery in couples actively trying to conceive.
Association between the improvement of OS and sperm DNA fragmentation levels after varicocele repair with ART outcomes.	Prospective cohort studies including infertile men with increased OS markers and sperm DNA fragmentation levels undergoing varicocele repair. Participants should wait at least 3 months after surgery for ART treatments and should be followed up until the end of each treatment.

8-OHdG: 8-hydroxy-2′-deoxyguanosine; ART: assisted reproduction techniques; MDA: malondialdehyde; OS: Oxidative stress; ROS: Reactive oxygen species; SA: semen analysis; TAC: total antioxidant capacity;

## CONCLUSIONS

A growing evidence body supports oxidative stress and sperm DNA damage as critical factors in the pathophysiology of varicocele-related infertility. However, the pathways by which varicocele causes oxidative stress are not fully understood. In some men with varicocele, defense mechanisms against excessive ROS production are defective, leading to spermatogenesis impairment and subsequent infertility. Sperm DNA fragmentation is one of the adverse effects of varicocele-induced oxidative stress; elevated sperm DNA fragmentation levels decrease the chance of natural conception and ART success. Varicocele repair may restore the balance between reactive oxygen species and antioxidants, alleviating sperm DNA damage and improving the likelihood of natural and assisted pregnancy in men with palpable varicocele and infertility. These findings have resulted in changes to clinical practice guidelines, incorporating sperm DNA fragmentation testing for infertile men with palpable varicocele and varicocelectomy in cases of elevated sperm DNA fragmentation levels. Gaps in knowledge exist, including understanding the mechanisms behind increased ROS production and sperm DNA fragmentation in men with varicocele. In addition, the impact of varicocele grade and laterality on OS and sperm DNA fragmentation, as well as the effect of improved OS and sperm DNA fragmentation levels in pregnancy and live birth rates after varicocelectomy, are still unclear and deserve further investigation.
